# Bacteriological Profile of Diabetic Wounds at Laquintinie Hospital Douala and the Antimicrobial Potential of Spiced Fish (*Fontitrygon margarita*) Liver Oil Against Multiresistant Isolates

**DOI:** 10.1155/bmri/3158942

**Published:** 2025-06-01

**Authors:** Estelle Djeukoua Djeuya, Joseph Paul Marius Koualiagnigni, Fabrice Fabien Dongho Dongmo, Sammuel Raymond Tchabong, Sorelle Daina Nanhou Nkepndep, Boris Simo Noutsa, Alix G. T. Tchoupe, Josiane Essola, Rebecca Madeleine Ebelle Etame, Modeste Lambert Sameza

**Affiliations:** ^1^Estuary Academic and Strategic Institute, University of Douala, Douala, Cameroon; ^2^Department of Biochemistry, Faculty of Science, University of Douala, Douala, Cameroon; ^3^Department of Processing and Quality Control of Aquatic Products, Institute of Fisheries and Aquatic Sciences, University of Douala, Douala, Cameroon; ^4^Laquintinie Hospital of Douala, Douala, Cameroon

**Keywords:** antibiotic resistance, antioxidant activity, diabetic wound infections, *Fontitrygon margarita* liver oil, natural antibacterial agents, spices and bioactive compounds

## Abstract

This study investigates the bacteriological profile, antibiotic susceptibility, and potential natural treatment for diabetic wound infections at Laquintinie Hospital in Douala, Cameroon. Over 2 months (June to July 2024), 75 diabetic patients with wounds were analyzed, revealing a 73.08% infection rate. The most common bacterial isolates were *Staphylococcus aureus* (54.38%), *Klebsiella pneumoniae* (40.35%), and *Escherichia coli* (29.82%), with significant antibiotic resistance observed. Imipenem (82.65% sensitivity) and gentamicin (72.88% sensitivity) were the most effective antibiotics, while amoxicillin–clavulanic acid showed the highest resistance rate (46.94%). This study also evaluated the antibacterial and antioxidant efficacy of *Fontitrygon margarita* liver oil, particularly when extracted with spices such as *Monodora myristica* and *Zingiber officinale*. The oil demonstrated significant antibacterial activity against multiresistant bacterial isolates, with minimum inhibitory concentrations ranging from 16 to 128 mg/mL. Incorporating spices during extraction enhanced the oil's antibacterial activity, showing synergistic effects in 58.33% of cases. The oil also exhibited strong antioxidant properties, with improvements noted in DPPH, ABTS, and FRAP assays when spices were added, highlighting the highest efficacy with *M. myristica* and *Z. officinale* extracts. These findings suggest that *F. margarita* liver oil, especially when combined with spices, could serve as a valuable natural alternative for managing diabetic wound infections, addressing the challenges posed by antibiotic resistance and oxidative stress. Further research and clinical trials are recommended to validate these results and explore their practical applications.

**Summary**

• High prevalence of diabetic wound infections in Douala.
• High resistance of commonly involved bacterial species to common antibiotics.
• *Fontitrygon margarita* liver oil shows strong antibacterial and antioxidant properties.
• Spices enhance the yield and bioefficacy of bioactive compounds in this oil.
• *Monodora myristica* and *Zingiber officinale* improve the effectiveness of the oil.
• Natural products offer a promising alternative for diabetic wound infections.

## 1. Introduction

Diabetes is the most common metabolic disease and a major public health issue worldwide due to its rising prevalence [1]. In Cameroon in particular, it affects over 567,000 people and leads to severe complications, increased morbidity, mortality, and high healthcare costs [2]. Complications include diabetic ketoacidosis, cardiovascular disease, neuropathy, nephropathy, retinopathy, and wounds [3]. Diabetes impairs wound healing due to reduced blood flow, neuropathy, and weakened immune function. Common complications are infections, chronic wounds, and foot ulcers, which can lead to amputation if untreated [4, 5]. Globally, diabetics are prone to “unhealable wounds,” which foster bacterial growth and resistance to treatments, leading to multidrug resistance. Multiresistant bacterial isolates or strains develop resistance to at least one agent in three or more antimicrobial categories, making them especially challenging to treat [5].

Effective diabetes management can reduce the risk of wound complications through regular monitoring, blood sugar control, a healthy lifestyle, and proper wound care [5, 6, 7]. Antibiotics are essential for treating infections and preventing their spread. Proper use in combination with good wound care and blood sugar management improves clinical outcomes. Identifying bacteria and performing antibiograms are necessary for adequate prescription. In Cameroon, limited resources lead to the use of antibiotics without prescriptions, contributing to bacterial resistance [8, 9]. Exploring antibiotic alternatives is also crucial due to issues related to high toxicity, high cost, and low availability. Natural products are being explored due to their availability, low cost, easy application, and few side effects [10]. These products not only fight against microorganisms but also have antioxidant, anti-inflammatory, and immunomodulatory properties. They are organic compounds from various organisms, including plants, and are often consumed as functional or medicinal foods for their health benefits [10]. These foods provide essential nutrients and active ingredients that aid in disease treatment and overall health [11]. Researchers are now focusing on extracting these compounds at higher concentrations to maximize their benefits.

In this respect, omega-3 fatty acids, particularly eicosapentaenoic and docosahexaenoic acids, have significant antimicrobial and antibiofilm properties against various microbes, including drug-resistant isolates [12, 13]. They disrupt bacterial cell membranes and essential processes, leading to cell lysis and death [14]. These fatty acids also have anti-inflammatory effects and play roles in immune signaling [15]. Extracting these nutraceuticals can be challenging, so using sources like fatty fish is practical. Fish oils, rich in omega-3s, have been explored for their antimicrobial potential in Cameroon and globally [16]. They also contain beneficial compounds like vitamins, minerals, and phytocompounds [17]. Fish oil components aid in treating chronic wounds by reducing inflammation and promoting new tissue formation [16]. However, fish oil is prone to oxidation, which affects its quality and therapeutic benefits [18]. Adding natural antioxidants, such as spices rich in phenolics, can protect against oxidation and enhance antimicrobial properties [19, 20]. Phenolic compounds disrupt bacterial processes and have antioxidant, anti-inflammatory, and immunomodulatory effects [21]. Thus, spices are ideal for protecting fish oils from oxidation.

Fish production is vital in Cameroon, with studies on various species focusing on oil extraction yield, physicochemical characteristics, and antimicrobial activities [15]. From our recent research on fish, *Fontitrygon margarita* (Stingray), liver oil showed an extraction yield of 14.49%–16.9% and high omega-3 fatty acid content. The oil demonstrated antibacterial properties against food poisoning bacteria [22], suggesting its potential for managing diabetic wound infections. Additionally, incorporating spices like *Allium sativum* (garlic), *Monodora myristica* (calabash nutmeg), and *Piper nigrum* (black pepper) prior to extraction reduced oxidability and oxidation and maintained the oil's antibacterial activities [20].

Moreover, diabetic wounds are particularly prone to infections and delayed healing due to oxidative stress, which arises from an imbalance between reactive oxygen species (ROS) and antioxidants [23]. This oxidative stress damages cells and tissues, impairs the healing process, and weakens the immune response, facilitating bacterial colonization and biofilm formation that are resistant to antibiotics. The persistent presence of ROS and inflammation contributes to the chronic nature of diabetic wounds [24]. Reducing oxidative stress through antioxidants, such as bioactive compounds, can enhance wound healing by neutralizing ROS and promoting tissue repair [23]. Therefore, understanding this relationship is crucial for developing effective treatment strategies for diabetic wound management. To our knowledge, no similar study has been conducted according to the available literature.

This research combines traditional medicine with modern microbiological techniques to develop a natural therapeutic option for managing diabetic wound infections. It investigates the bacteriological profile of diabetic wounds, antibiotic susceptibility of the pathogens, and the potential of spiced *F. margarita* liver oil as a natural agent. The spices selected for this study, such as *A. sativum*, *M. myristica*, *P. nigrum*, and *Zingiber officinale* (ginger), are readily available in Cameroon and are known for their bioactive properties. *A. sativum* provides antimicrobial and antioxidant activities due to allicin, while *M. myristica* offers phenolic compounds and flavonoids with similar benefits. *P. nigrum* enhances the bioavailability of other compounds through piperine, and *Z. officinale* provides anti-inflammatory, antioxidant, and antimicrobial properties due to gingerol [19, 20, 22]. Incorporating these spices before extracting *F. margarita* liver oil enhances its bioactive compound content, improving its antibacterial and antioxidant efficacy and providing a promising natural therapeutic option for diabetic wound infections.

Therefore, this study was conducted at the Laquintinie Hospital in Douala (HLD) to analyze the bacteriological profile of germs in diabetic wounds and their antibiotic susceptibilities. Additionally, we evaluated the efficacy of *F. margarita* liver oil, extracted with spices (*A. sativum*, *M. myristica*, *P. nigrum*, and *Z. officinale*), in managing diabetic wound infections, focusing particularly on its capacity to combat multiresistant bacterial isolates and mitigate oxidative stress.

## 2. Materials and Methods

### 2.1. Study Design

This cross-sectional study investigates the bacteriological profile of diabetic wounds, the antibiotic susceptibility of pathogens, and the potential antibacterial and antioxidant effects of *F. margarita* liver oil extracted with spices. Diabetic patients with wound were recruited from HLD, and informed consent was obtained. Wound swabs were collected and transported to the HLD microbiology laboratory for analysis. The liver of *F. margarita* was collected at Douala Youpwe market and processed for oil extraction at the University of Douala's Valorization and Quality Control Laboratory in Yabassi, with and without spices. The oil's antibacterial activity was evaluated at the University's Biochemistry Lab against multiresistant bacterial isolates, and its antioxidant activity was also assessed there. Data were analyzed to determine the bacteriological profile, antibiotic susceptibility patterns, and the efficacy of the spiced liver oil.

### 2.2. Bacteriological Profile and Antibiotic Susceptibility of Diabetic Wound Microorganisms

#### 2.2.1. Study Location, Period, and Population

This study was conducted at HLD due to its advanced technical platform, diverse range of services, high patient volume, and convenient accessibility. The study took place over a 2-month period from June to July 2024. The study population consisted of diabetic patients with clinically diagnosed wounds who were admitted to and/or hospitalized in various departments of the hospital during this time.

#### 2.2.2. Enrollment Criteria

The inclusion criteria for this study encompassed diabetic patients with clinically diagnosed wounds who provided informed consent. Exclusion criteria included patients with nondiabetic wounds, those who did not provide informed consent, and patients returning to the hospital for follow-up who had previously been included in the study.

#### 2.2.3. Ethical Considerations

The study strictly adhered to established medical research principles. Data collection forms were coded to ensure anonymity, and all information remained confidential. The study posed no risk to patients. Participants were fully informed about the study's objectives, purpose, and benefits before providing informed consent. Ethical clearance (No. 4328CEI-Udo/07/2024/M) was obtained from the Institutional Ethics Committee of the University of Douala, and a collection authorization (No. 057/ARC/MINSANTE/DHL/SG) was secured from HLD.

#### 2.2.4. Sampling

The sample size was determined using the Lorentz formula: *n* = *z*^2^ × *p* × (1 − *p*)/m^2^, where *n* is the sample size, *z* is the confidence level (1.96), *p* is the estimated proportion (13% from Yadav et al. [5]), and *m* is the margin of error (5%). The calculated sample size was 173. To account for unsatisfactory responses or errors, this number was increased by 10%, resulting in a final sample size of 190. The study utilized a probabilistic sampling method, specifically convenience sampling, where every consented diabetic patient presenting at the hospital was included. Despite the planned sample size, only 75 diabetic patients with wounds consented to participate during the study period.

#### 2.2.5. Analysis of the Bacteriological Profile of Germs in Diabetic Wounds and Their Antibiotic Susceptibilities

##### 2.2.5.1. Data Collection

We gathered sociodemographic and clinical data, including age, sex, diabetes type, comorbidities, wound topography, wound mechanism, and macroscopic appearance. Wound samples were collected for bacteriological analyses, including bacterial isolation, identification, and antibiotic susceptibility testing.

##### 2.2.5.2. Sample Collection

Wounds were cleaned with sterile gauze soaked in saline to remove commensal flora and necrotic tissues. Sampling was done using two labeled swabs over a 1 cm^2^ surface in a Z-shaped motion. One swab detected microorganisms, and the other isolated and identified them. The wound's macroscopic appearance was noted during sampling.

##### 2.2.5.3. Detection of Microorganisms in the Samples

Detection was done in two states: fresh and stained. In the fresh state, samples were observed between a slide and coverslip with physiological water, using 10× and 40× objectives (Microscope Olympus, Japan) to determine the presence and mobility of microorganisms [25]. For the stained state, Gram staining (Hardy Diagnostics, United States) was used. Smears were dried, stained with crystal violet, and fixed with iodine. Decolorization with ethanol distinguished Gram-positive and Gram-negative bacteria. A counterstain with fuchsin stained Gram-negative bacteria pink, while Gram-positive bacteria retained the violet color. Rinsing with water after each step was crucial to stop the reaction and remove excess dye. Prolonged decolorization was avoided to prevent decolorizing Gram-positive bacteria. Microscopic observation of stained smears allowed us to note bacterial shapes (cocci, bacilli, and coccobacilli), grouping (clusters and chains), and differentiate Gram-positive (violet) from Gram-negative (pink).

##### 2.2.5.4. Isolation and Identification of Bacteria

Several culture media, including eosin methylene blue (EMB) (Merck, Germany), Chapman Agar (Sigma-Aldrich, Germany), and fresh blood agar (GS) (SSI Diagnostica, United States), were prepared and inoculated to grow different bacteria. Media were prepared 3 days before use, following manufacturers' instructions, and underwent sterility and fertility tests. Sterility was confirmed if no bacterial growth was observed after 48 h at 37°C, using sterile media without any bacterial inoculation as a negative control. Fertility was confirmed if growth was observed after 24 h with specific isolates: *Staphylococcus aureus* on Chapman, *Escherichia coli* on EMB, and *Streptococcus* on GS, serving as positive controls.

Isolation was done using the quadrant streak method around a flame. Inoculated media were incubated for 24 h at 37°C. Bacterial isolates on Chapman and GS media were identified by colony appearance, color change, hemolysis, and Gram staining. Isolates on EMB medium were identified using the Api 20 E gallery (bioMérieux, France), a standardized system for identifying Enterobacteriaceae and other nonfastidious Gram-negative bacilli. The bacterial inoculum was prepared to a concentration of 0.5 McFarland. Labeled Api 20 E galleries were filled with the inoculum, and specific microtubes were filled with immersion oil. Galleries were incubated at 37°C for 24 h, and reactions were read using a table and analytical catalog. Gram staining was performed on different colonies for further differentiation and classification.

##### 2.2.5.5. Study of the Susceptibility of Isolated Bacteria to Common Antibiotics

The antibiogram process began with preparing the bacterial inoculum. A pure bacterial colony was emulsified in physiological water to match 0.5 McFarland. The Mueller–Hinton medium (Merck, Germany) was inoculated by swabbing with the bacterial inoculum. Antibiotic disks were placed on the medium, which was incubated for 24 h at 37°C. The four antibiotics used in this study were among the most commonly used in Cameroon [26]: two from the beta-lactam family, specifically one penicillin (amoxicillin–clavulanic acid) and one carbapenem (imipenem); one from the aminoglycoside family (gentamicin); and one from the quinolone family (ciprofloxacin). Controls were included to validate the accuracy of the antibiotic susceptibility tests. Negative controls (sterile media without any antibiotic disks) and positive controls (media inoculated with known resistant or sensitive strains) ensured the tests' validity and reliability.

After incubation, inhibition zone diameters around the disks were measured and interpreted according to the sensitivity scale (sensitive–intermediate–resistant) based on CA-SFM/EUCAST [27] and CLSI [28] recommendations. For amoxicillin–clavulanic acid (20–10 *μ*g, Medopharm, India), sensitivity was ≥ 23 mm, intermediate was 16–23 mm, and resistant was < 16 mm. For imipenem (10 *μ*g, Merck & Co., Singapore), sensitivity was ≥ 24 mm, intermediate was 17–24 mm, and resistant was < 17 mm. For gentamicin (10 *μ*g, Suanfarma, Spain), sensitivity was ≥ 17 mm, intermediate was11–17 mm, and resistant was < 11 mm. For ciprofloxacin (5 *μ*g, Bayer AG, Germany), sensitivity was ≥ 25 mm, intermediate was 22–25 mm, and resistant was < 22 mm.

### 2.3. Efficacy of *F. margarita* Liver Oil Extracted With Various Spices in Managing Diabetic Wound Infections

The livers of freshly caught *F. margarita* fish (1.0 kg) were collected from the Douala Youpwe market in November 2023. These fish were identified by ichthyologists from the Fisheries Resources Laboratory of the Institute for Fisheries and Aquatic Sciences. The livers were carefully removed by skilled fish cleaners and placed in ice boxes for transport to the Valorization and Quality Control Laboratory at the University of Douala in Yabassi, Cameroon.

The bulbs of *A. sativum*, hulls of *M. myristica*, seeds of *P. nigrum*, and rhizomes of *Z. officinale* were purchased from the local Youpwe market, dried, and ground into powder. The botanical identification of the plants was performed by a local botanist and confirmed at the Cameroon National Herbarium, where the voucher specimens were conserved under the reference numbers 44810/HNC (*A. sativum*), 2949/SFR-Cam (*M. myristica*), 25818/SFR/Cm (*P. nigrum*), and 14757/SRF/Cam (*Z. officinale*). A portion of the powders was used for oil extraction, while the remaining powder underwent aqueous extraction to quantify key antioxidant constituents, including total phenolic content (TPC) and flavonoid content, following the protocol in Simo et al. [20]. Briefly, approximately 25 g of each spice powder was combined with sterilized distilled water to yield a 100 mL solution, creating an aqueous extract with a concentration of 25% *w*/*v*. This mixture was allowed to stand at room temperature (28°*C* ± 2°C) for 48 h in a sterile flask, with regular shaking. The solution was then centrifuged at 3000 rpm for 10 min and filtered through Whatman No. 1 filter paper. The filtrate was concentrated using a rotary evaporator set to a temperature range of 55°C–60°C. The extracts were subsequently evaluated for their TPC and flavonoid content. The TPC was determined using the Folin–Ciocalteu method. In this procedure, 20 *μ*L of the appropriately diluted spice extract (100 mg/mL) was combined with 0.2 mL of Folin–Ciocalteu reagent (Merck, Germany) and 2 mL of distilled water in a test tube. This mixture was incubated at room temperature for 3 min. Subsequently, 1 mL of a 20% sodium carbonate solution was added, and the mixture was reincubated at room temperature for an additional 2 h. A blank was prepared using 400 *μ*L of distilled water in place of the sample. The absorbance of the resultant blue mixture was measured at 765 nm using a quartz cuvette. Gallic acid was used as the standard, and the TPC was quantified as milligrams of gallic acid equivalents per gram. Total flavonoid content was measured using an aluminum chloride complex assay. The procedure started by mixing 100 *μ*L of each diluted extract (100 mg/mL) with 100 *μ*L of 5% sodium nitrate. This mixture was allowed to stand for 6 min. Then, 150 *μ*L of a 10% aluminum chloride solution (Merck, Germany) was added, and the solution was left to stand for an additional 5 min. Subsequently, 200 *μ*L of a 1 M sodium hydroxide solution (Merck, Germany) was added sequentially. The absorbance of the resulting reaction mixture was measured at 510 nm using a UV spectrophotometer. The total flavonoid content of the extracts was then calculated in terms of quercetin equivalents (expressed as milligrams of quercetin equivalents per gram). All procedures were performed in triplicate.

Bacterial isolates used in this phase of the study were selected based on their antibiotic susceptibility profiles from the first phase. Specifically, isolates demonstrating multiresistance, defined as resistance to at least three out of the four antibiotics tested, were chosen.

#### 2.3.1. Extraction Process of *F. margarita* Liver Oil

The extraction of *F. margarita* liver oil followed the method described by Simo et al. [20]. Freshly obtained livers were sliced, combined, and divided into five batches of 200.0 g each for different extraction processes. Some batches included spice powders, while others did not. Spices were added at 2000 ppm, which was determined to be the optimal proportion according to our previous study. The mixtures were stirred for 10 min before oil extraction using the exudation technique. Briefly, samples were wrapped in aluminum foil to prevent moisture loss and potential contamination. The wrapped samples were placed in a preheated oven set at 95°C for 10 min. After heating, the samples were carefully removed from the oven, and the exuded oil was collected by gently unwrapping the aluminum foil and allowing the oil to drain into a collection vessel. Anhydrous sodium sulfate (Sigma-Aldrich, United States) was used to remove all traces of moisture. The oil was then weighed, transferred to dark vials, and stored at 4°C until use. The oil was then weighed, and the yield of the samples was calculated using the following formula:

 $$\begin{array}{c} \text{Yield }\left(\%\right)=\left(\frac{\text{Mass of extracted oil}}{\text{Mass of sample}}\right)\times 100. \end{array}$$

A portion of each oil sample was analyzed for its phytochemical composition, focusing on TPC and flavonoid content. These analyses adhered to the previously outlined protocols, with the notable adjustment of using the oil directly in its undiluted form. Simultaneously, total carotenoid content was evaluated by photometry at 446 nm using an iCheck Carotene Photometer (BioAnalyt GmbH, Germany), following the protocol described by Dongho et al. [29]. Briefly, a 20 *μ*L sample of oil was diluted with 2 mL of hexane. After vigorous shaking, the mixture was read using the photometer, which provided the carotenoid content of the solution in milligrams per liter. The carotenoid content of the oil was then calculated by considering the dilution factor.

The remaining oil samples were used to determine antibacterial and antioxidant activities using standardized methods.

#### 2.3.2. Assessment of Antibacterial Activity of *F. margarita* Liver Oil Against Multiresistant Bacterial Isolates

Bacterial isolates were prepared by culturing them on appropriate agar media under optimal conditions specific to their species. Bacterial cultures were stored at 4°C on agar slants for short-term maintenance. Before each assay, cultures were revived by streaking onto fresh agar plates and incubating under the same conditions. A single colony from these plates was then used to inoculate broth cultures, incubated overnight at 37°C.

The antibacterial properties of the oil were assessed using the broth microdilution method in 96-well microplates, as described by Simo et al. [20], adhering to the CLSI standards [30].

Oils were prepared as stock solutions at 1024 mg/mL in a 5% Tween 80 solution. Mueller–Hinton agar culture was used to prepare the bacterial isolate inoculum. Bacterial suspensions were created with a concentration of approximately 1.5 × 10^8^ CFU/mL, equivalent to a McFarland turbidity standard No. 0.5. The inoculum was then diluted to a final concentration of 1.5 × 10^6^ CFU/mL.

Each well of the microplate was filled with 100 *μ*L of culture broth. Then, 100 *μ*L of oil was added to the top wells, and a series of two-fold dilutions was performed to achieve final concentrations ranging from 2 to 1024 mg/mL. Each well was further diluted with 100 *μ*L of inoculum. The plates were incubated at 35°C for 18 h. Growth was monitored using 0.2 mg/mL p-iodo tetrazolium chloride (Sigma-Aldrich, United States). Viable bacteria changed the yellow dye to pink. The lowest concentration of oil at which no visible color change was noted was considered the minimum inhibitory concentration (MIC).

To assess the interaction between oil and spices, the fractional inhibitory concentrations (FICs) were calculated as follows [31]:

 $$\begin{array}{c} \text{FIC}=\frac{\text{MI}{\text{C}}_{\text{Oil⁣extracted⁣with⁣spice}}}{\text{MI}{\text{C}}_{\text{Oil⁣extracted⁣without⁣spice}}}. \end{array}$$

Interactions were classified based on FIC values as synergistic (FIC ≤ 0.5), additive (0.5 < FIC ≤ 1), indifferent (1 < FIC ≤ 4), and antagonistic (FIC > 4).

#### 2.3.3. Evaluation of Antioxidant Activity of Extracted Oil

The antioxidant activity of the extracted oils was evaluated in vitro through free radical scavenging activity (RSA) and reducing power, adapted from protocols used by Christodouleas et al. [31], Amorati et al. [32], and Song et al. [33]. Free RSA was assessed using the DPPH and ABTS assays. The DPPH assay measures the antioxidant capacity by evaluating the ability to neutralize the DPPH radical (DPPH•), while the ABTS assay assesses the ability to quench the ABTS radical cation (ABTS•+). The reducing power was determined using the FRAP (Merck, Germany) assay, based on the reduction of Fe(III) to Fe(II) in the presence of the oils. Ascorbic acid was used as a reference standard.

To prepare the samples, an initial dilution was made by dissolving 2 mg of the oil sample in 2 mL of water with Tween 80 as an emulsifier. From this initial solution, a series of further dilutions was prepared, ranging from 1 to 500 *μ*g/mL.

For the DPPH assay, 200 *μ*L from each dilution was combined with 200 *μ*L of DPPH solution (Merck, Germany) and 600 *μ*L of methanol (Merck, Germany). The mixtures were then incubated in the dark for 30 min. After the incubation period, the absorbance was measured at 517 nm (Spectrophotometer Thermo Fisher Scientific model AQUAMATE 7100, United States). The RSA was calculated, and the IC50 value, representing the concentration of antioxidant needed to scavenge 50% of the DPPH radicals, was determined [31, 32, 33].

As for the ABTS assay, 200 *μ*L of oil was mixed with 1800 *μ*L of the ABTS solution (Sigma-Aldrich, United States), incubated in the dark for 15 min, and absorbance was measured at 734 nm against a blank. The inhibition percentage was calculated, and the IC50 value was determined [31, 32, 33].

To evaluate the reducing power of a sample, 1.25 mL of phosphate buffer (0.2 M, pH 6.6) and 1.25 mL of a 1% potassium ferricyanide solution [K_3_Fe(CN)_6_] (Merck, Germany) were added to 50 *μ*L of the sample at various concentrations. The mixture was incubated at 50°C for 20 min to allow for the reduction reaction to occur. Following incubation, 1.25 mL of a 10% trichloroacetic acid solution (Merck, Germany) was introduced to precipitate any proteins present in the mixture. After centrifugation at 3000 rpm for 10 min (Centrifuge SIGMA 2-6E, Germany), the supernatant was collected, and to it, 1.25 mL of distilled water and 250 *μ*L of 0.1% FeCl_3_ (Merck, Germany) were added. The absorbance of the resulting solution was measured at 700 nm against a blank. The results were expressed as the effective concentration at 50% (EC50), which represents the concentration of the sample required to achieve an absorbance of 0.5 at 700 nm [32, 33].

### 2.4. Statistical Analyses

Statistical analysis was conducted using GraphPad Prism software Version 5.9. Qualitative variables were described using frequencies and percentages, while quantitative variables were expressed as mean and standard deviation. Percentages were compared using Pearson's chi-squared test. One-way ANOVA, followed by Tukey's post hoc tests, was used to compare means. A *p* value of less than 0.05 was considered statistically significant.

## 3. Results and Discussion

During this study, 358 diabetes patients were received at HLD between the months of June and July 2024. This prevalence aligns with global trends, as diabetic foot ulcers affect 15%–25% of diabetic patients [34]. A meta-analysis by Zhang et al. [35] highlighted the global burden of diabetic wounds. Our findings emphasize the need for effective prevention and management strategies. Regional studies, such as those by Tsaffo et al. [4] in Cameroon, report similar prevalence rates and challenges in managing diabetic wounds due to limited healthcare resources and high infection rates. Among the patients, 75 had wounds (20.95% prevalence), with 72 having a single wound and three having multiple wounds, indicating more severe complications. The analysis was then performed on 78 biological samples.

### 3.1. Characteristics of the Participants

As shown in Table 1, males were predominant (61.33%), with a sex ratio of 1.58 in favor of men, likely due to less frequent medical care and higher trauma exposure [36]. Participants' ages ranged from 29 to 82 years, with the most represented group being those over 60 (50.66%) and the least under 41 (10.67%). The average age was 59.54 years, reflecting the natural aging process that promotes diabetes and its complications [37].

Most participants had Type 2 diabetes (88.00%), with 60.00% presenting comorbidities like hypertension (38.67%), obesity (10.66%), stroke (10.66%), kidney diseases (2.67%), and HIV (2.67%). These data align with global trends, showing Type 2 diabetes as the most common, often due to aging, sedentary lifestyles, and being overweight [1, 36]. Wounds were mostly located on the feet (61.50%) and hands (14.10%), with fewer on the buttocks (12.80%), thighs (10.30%), and back (1.60%). Foot wounds are common due to mechanical constraints and initial degenerative complications [4, 5]. Wounds appeared spontaneously (42.31%), from trauma (38.46%), medical care (12.82%), or hygiene activities (6.41%), often due to degenerative complications [4, 5]. Macroscopic examination shows that most wounds were purulent and necrotic (34.62%), with others being necrotic (30.77%), purulent (12.82%), or without suppuration or necrosis (21.79%). These challenging wounds often require aggressive interventions like debridement and antibiotics [5, 6, 7].

### 3.2. Bacteriological Profile of Germs in Diabetic Wounds and Their Antibiotic Susceptibilities

#### 3.2.1. Prevalence of Diabetic Wound Infection

This study revealed that 57 wounds over the 78 analyzed were infected, representing 73.08% of the analyzed wounds. Table 1 shows that age was a significant factor (*p* = 0.039), with higher infection rates among patients aged 41–60 years (86.21%) and lower rates among those under 41 years (44.44%). Older diabetic patients are more susceptible to infections due to declining immune function and longer diabetes duration [38]. Infection rates did not vary significantly by sex, though men (78.72%) were more affected than women (64.57%).

Clinical factors significantly influenced diabetic wound infection rates. Higher infection rates were found for foot wounds (93.75%) due to poor circulation, neuropathy, and constant pressure [39]. Wounds from trauma (86.67%) or spontaneous occurrence (84.85%) were more infected (*p* = 0.0001), as traumatic wounds are often severe and exposed to contaminants [40]. Purulent and necrotic wounds were most infected (*p* < 0.0001), indicating severe infection and poor healing conditions [41]. Infection rates did not vary significantly by diabetes type, though Type 2 diabetic patients (76.47%) were more affected than Type 1 (50%). Comorbidities like cardiovascular disease and renal impairment increased infection risk (76.60% vs. 67.74%). All the two HIV-infected patients had infected wounds, highlighting the increased infection risk in immunocompromised patients [38].

#### 3.2.2. Profile of Isolated Germs

Table 2 shows the frequency of bacterial species isolated from 57 infected wounds. Gram-negative bacteria (*Klebsiella pneumoniae*, *E. coli*, *Proteus mirabilis*, *Pseudomonas aeruginosa*, *Morganella morganii*, *Enterobacter aerogenes*, and *Acinetobacter* spp.) were found in 60.21% of samples, while Gram-positive bacteria (*S. aureus*, *β*-hemolytic *Streptococcus*, and *Staphylococcus epidermidis*) were in 39.79% of samples. The prevalence of Gram-negative bacteria, including *K. pneumoniae* and *E. coli*, aligns with other studies highlighting their antibiotic resistance and ability to thrive in compromised tissues [7, 41]. However, Tsaffo et al. [4] noted a predominance of Gram-positive bacteria in Cameroon. The difference in bacterial prevalence could be due to variations in the study populations, environmental factors, or differences in healthcare settings [42].

Ten bacterial species were isolated, with *S. aureus* found in 54.38% of wounds, followed by *K. pneumoniae* (40.35%) and *E. coli* (29.82%). *S. aureus* commonly infects diabetic wounds by forming biofilms that shield it from the immune system and antibiotics [42]. *K. pneumoniae* and *E. coli* are prevalent due to their virulence factors and antibiotic resistance [43]. Other bacteria included *P. mirabilis* (10.53%), *β*-hemolytic *Streptococcus* (8.77%), *P. aeruginosa* (8.77%), *M. morganii* (7.02%), *E. aerogenes* (5.26%), *S. epidermidis* (5.26%), and *Acinetobacter* spp. (1.75%). The presence of these bacteria indicates a diverse microbial environment in diabetic wounds. *P. aeruginosa* is known for its resistance to multiple antibiotics and ability to form biofilms, complicating treatment. Studies by Gardner et al. [44] and James et al. [45] emphasize the complexity of microbial communities in chronic wounds. Tsaffo et al. [4] also found *S. aureus* as the most isolated germ (47.61%), followed by *P. aeruginosa* (23.80%), reflecting differences in microbial environments or patient demographics [7, 42].

Table 2 also shows that 38 wounds (66.67%) were polymicrobial, while 19 (33.33%) were monomicrobial. Polymicrobial infections complicate treatment and prolong healing [42]. The most common monomicrobial pathogen was *K. pneumoniae* (*n* = 9), known for its virulence and antibiotic resistance, particularly in immunocompromised patients [7, 41, 43]. In polymicrobial wounds, 61.4% were bimicrobial, with frequent associations between *S. aureus* and *E. coli* (*n* = 8), *S. aureus* and *K. pneumoniae* (*n* = 4), or *S. aureus* and *P. mirabilis* (*n* = 4). These combinations lead to more severe infections due to synergistic effects [7, 43]. Less common were associations between two Gram-negative bacteria or a Gram-positive bacterium other than *S. aureus* and another bacterium, suggesting specific environmental or host factors [7, 43, 44]. Trimicrobial wounds were less common (5.27%), with *S. aureus* frequently involved, highlighting its role as a key pathogen due to biofilm formation and antibiotic resistance [6, 7, 45].

#### 3.2.3. Antibiotic Sensitivity of Implicated Germs

Table 3 shows the susceptibility of bacterial isolates to antibiotics. The most sensitive species were *β*-hemolytic *Streptococcus* to amoxicillin–clavulanic acid (80%), *S. aureus* to imipenem (100%), *S. epidermidis* to gentamicin (100%), and *β*-hemolytic *Streptococcus* to ciprofloxacin (80%). Amoxicillin–clavulanic acid inhibits beta-lactamase enzymes, which are effective against *β*-hemolytic *Streptococcus*. Imipenem, a broad-spectrum carbapenem, works against many Gram-positive and Gram-negative bacteria, including resistant *S. aureus*. Gentamicin, an aminoglycoside, is effective against various Gram-positive and Gram-negative bacteria, especially in skin and soft tissue infections. Ciprofloxacin, a fluoroquinolone, targets a wide range of bacteria, including *β*-hemolytic *Streptococcus* [5, 6, 7, 41, 43, 44, 46, 47].

The most resistant species were *P. aeruginosa* to amoxicillin–clavulanic acid (100%), *M. morganii* to imipenem (75%), *β*-hemolytic *Streptococcus* to gentamicin (80%), and *S. epidermidis* to ciprofloxacin (66.66%). *P. aeruginosa* shows intrinsic resistance to many antibiotics, including beta-lactams, due to efflux pumps and beta-lactamase production. Resistance to carbapenems like imipenem can occur due to carbapenemase production or changes in porin channels in bacteria like *M. morganii*. Resistance to aminoglycosides like gentamicin can occur due to modifying enzymes that inactivate the antibiotic. Similarly, resistance to fluoroquinolones like ciprofloxacin can occur due to mutations in DNA gyrase or efflux pump mechanisms [5, 6, 7, 41, 43, 44, 46, 47].

Overall, the germs were more sensitive to imipenem (82.65%) and more resistant to amoxicillin–clavulanic acid (46.94%). Imipenem is effective against many resistant isolates, making it valuable for severe infections. The high resistance to amoxicillin–clavulanic acid may result from its extensive use, leading to resistant isolates [5, 6, 7, 41, 46, 47]. Gram-negative bacteria were more sensitive to gentamicin (72.88%) and Gram-positive bacteria to imipenem (100%). Gentamicin is effective against many Gram-negative bacteria, particularly those causing skin and soft tissue infections. Imipenem's broad-spectrum activity makes it highly effective against Gram-positive bacteria, including resistant isolates [6, 7, 46]. Regarding resistance, Gram-negative bacteria were more resistant to amoxicillin–clavulanic acid (69.49%) and Gram-positive bacteria to Ciprofloxacin (17.95%). Gram-negative bacteria's resistance is likely due to beta-lactamase production, while Gram-positive bacteria's resistance to fluoroquinolones like ciprofloxacin can occur due to mutations in DNA gyrase or efflux pump mechanisms [5, 6, 7, 41, 46, 47].

The high resistance rates to commonly used antibiotics like amoxicillin–clavulanic acid highlight the need for careful antibiotic stewardship to prevent the spread of resistant isolates. Understanding the specific sensitivity and resistance profiles of bacterial isolates can help tailor antibiotic therapy to individual patients, improving treatment outcomes and reducing resistance risk. These findings underscore the importance of selecting appropriate antibiotics based on susceptibility testing. The effectiveness of imipenem and gentamicin against a broad range of bacteria suggests they could be valuable options for treating diabetic wound infections, but their use should be carefully managed to prevent resistance [5, 6, 7, 41, 43, 44, 46, 47].

Globally, the mechanisms of antibiotic resistance observed in this study can be categorized by the mode of action. Inhibition of cell wall synthesis involves antibiotics like penicillins (amoxicillin–clavulanic acid), cephalosporins, and carbapenems (imipenem), with resistance mechanisms including beta-lactamase production and altered penicillin-binding proteins. Protein synthesis inhibition includes aminoglycosides (gentamicin), macrolides, and tetracyclines, countered by target site modifications, efflux pumps, and enzymatic inactivation. Nucleic acid synthesis inhibition involves quinolones (ciprofloxacin) and rifamycins, with resistance mechanisms such as mutations in DNA gyrase or topoisomerase IV, efflux pumps, and plasmid-mediated genes. Cell membrane disruption by polymyxins faces resistance mechanisms like lipid A modifications and efflux pump overexpression. Antimetabolite activity involves sulfonamides and trimethoprim, with resistance resulting from overproduction and mutation of target enzymes and alternative metabolic pathways [5, 6, 7, 41, 43, 44, 46, 47].

### 3.3. Efficacy of *F. margarita* Liver Oil Extracted With Various Spices in Managing Diabetic Wound Infections

#### 3.3.1. Phytochemical Composition of Spice Extracts and of *F. margarita* Liver Oil

##### 3.3.1.1. Compositions of Spice Extracts

**Figure 1:**
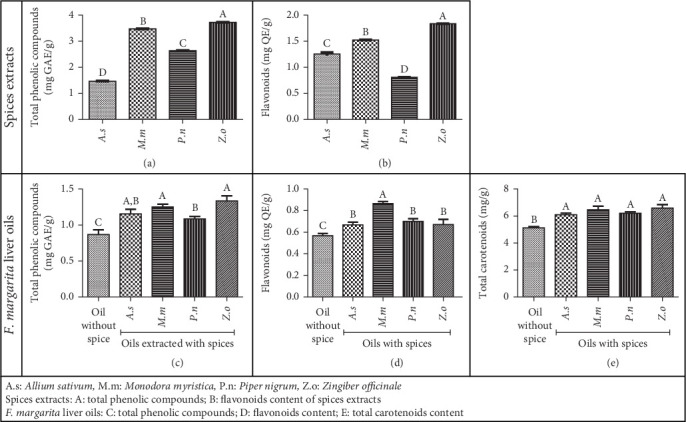
Phytochemical composition of spice extracts and of *Fontitrygon margarita* liver oil. Spice extracts: (a) total phenolic compounds; (b) flavonoids content of spice extract *F. margarita* liver oils; (c) total phenolic compounds; (d) flavonoid content; (e) total carotenoid content.

Figure 1 shows the TPC (Figure 1a) and flavonoid content (Figure 1b) of the aqueous extracts from the four spices used in this study. The TPC ranged from 1.46 to 3.72 mg GAE/g, with *Z. officinale* having the highest TPC, followed by *M. myristica* and *A. sativum* having the lowest. Flavonoid content varied from 0.81 to 1.84 mg QE/g, with *Z. officinale* having the highest, followed by *M. myristica* and *P. nigrum* having the lowest. These values align with previous studies [24, 48, 49, 50].

The presence of these bioactive compounds in spice extracts supports their efficacy in preventing oxidative reactions during the extraction and storage of *F. margarita* liver oil. Phenolic compounds are known for their antioxidant activity through mechanisms like free radical scavenging, metal chelation, and regeneration of other antioxidants [51]. These compounds can enhance the antioxidant activities of *F. margarita* liver oil extracts if they remain after extraction. Phenolic compounds also have broad-spectrum antimicrobial activities, useful against various bacterial infections, including multiresistant isolates. Their mechanisms include cell wall disruption, protein denaturation, DNA interference, and enzyme inhibition [52, 53, 54].

The bioactivity of phenolic compounds depends on their concentration in the spices, with higher concentrations generally correlating with stronger antioxidant and antimicrobial activities [53, 55]. The qualitative aspect is also crucial due to the diversity of phenolic compounds in the same source. Spices and fish oils are known to contain phenolic compounds [17], allowing for the presence of phenolics from various sources. Different phenolics, such as flavonoids and phenolic acids, have varying biological properties, and their combination can result in synergistic effects, enhancing overall biological activity [56, 57].

##### 3.3.1.2. Composition of *F. margarita* Liver Oil

The extraction yield observed *F. margarita* liver oil was 51.94% ± 1.92%, significantly higher than the 16.9% yield reported by Simo et al. [22]. This difference is likely due to the period of sample collection, which was in November for this work, but in April for Simo et al.'s work. Additionally, the age and size of the fish could have contributed to this variation. Figure 1 presents the TPC (Figure 1c), flavonoid content (Figure 1d), and total carotenoid content (Figure 1e) of oils extracted with and without spices. The oil extracted without spices had a significantly lower TPC (0.87 mg GAE/g) compared to oils extracted with spices (1.08–1.34 mg GAE/g), with the sample extracted with *P. nigrum* having the lowest TPC among the spiced oils. Similarly, the flavonoid content was lower in the oil extracted without spices (0.57 mg QE/g) compared to oils extracted with spices (0.67–0.86 mg QE/g), with the sample extracted with *M. myristica* showing the highest flavonoid content. These findings confirm the presence of substantial amounts of phenolic compounds, including flavonoids, in *F. margarita* liver oil, which could be partly responsible for its biological properties [17].

Incorporating powders of *A. sativum*, *Myristica fragrans*, *P. nigrum*, and *Z. officinale* into *F. margarita* liver before extraction increases the bioactive compound content in the oil. The variations in TPC and flavonoid levels among the different spices suggest that the type of spice used influences the amount of these compounds transferred to the oil. This transfer is influenced by the concentration of bioactive compounds in the spices, demonstrating a direct correlation between their levels in the spices and the resulting levels in the oil. The enhanced presence of phenolic compounds and flavonoids in the oil implies improved antioxidant and antimicrobial properties, enhancing the oil's stability and health benefits [58, 59].

The total carotenoid content of *F. margarita* liver oil was significantly lower in oil extracted without spices (5.16 mg/g) compared to samples extracted with spices (6.10–6.59 mg/g). Although there was no significant difference between the spices, oils extracted with *M. fragrans* and *Z. officinale* had slightly higher carotenoid contents compared to those with *A. sativum* and *P. nigrum*. This result confirms the presence of carotenoids in *F. margarita* liver oil, as in other fish oils [17]. Carotenoids are renowned for their antimicrobial properties through several mechanisms, including disrupting microbial membranes, generating ROS to induce oxidative stress, inhibiting key enzymes involved in microbial metabolism, and interfering with quorum sensing [60, 61]. They are also known for their antioxidant and anti-inflammatory properties, which significantly contribute to the biological activities of fish oils [62, 63]. The higher carotenoid content in oils extracted with spices suggests that spices play a crucial role in preserving carotenoids during the extraction process [58, 59].

Overall, these results underscore the potential of incorporating spices during the extraction process. This approach not only increases the bioactive compound content of the oil but also helps retain valuable fish bioactive compounds such as carotenoids, enhancing the oil's stability and health benefits.

#### 3.3.2. Antibacterial Activity of Extracted Oil Against Multiresistant Bacterial Isolates

A total of 15 bacterial isolates, representing seven species, were selected to assess their susceptibility to *F. margarita* liver oil. This selection included three isolates each of *E. coli* (Ec1, Ec2, and Ec3), *K. pneumoniae* (Kp1, Kp2, and Kp3), *P. aeruginosa* (Pa1, Pa2, and Pa3), and *S. aureus* (Sa1, Sa2, and Sa3). The other three species were represented by a single isolate each: *E. aerogenes* (Ea1), *M. morganii* (Mm1), and *P. mirabilis* (Pm1). The isolates Ec1, Ec2, Kp2, Kp3, Pa2, and Pa3 were resistant to three antibiotics except for ciprofloxacin, while the isolates Sa1, Sa2, and Sa3 were resistant to three antibiotics except for imipenem. The remaining six isolates were resistant to all four antibiotics.

Table 4 presents the antibacterial activities of *F. margarita* liver oil against the selected multiresistant bacterial isolates. The results indicate that oil extracted without spices exhibits antibacterial activity against all tested bacterial isolates, with MIC values ranging from 16 to 128 mg/mL. Among the 15 isolates tested, one isolate showed an MIC of 16 mg/mL, five isolates had an MIC of 32 mg/mL, eight isolates had an MIC of 64 mg/mL, and one isolate had an MIC of 128 mg/mL. Overall, these MIC values suggest that the seven bacterial species tested have relatively similar susceptibility to *F. margarita* liver oil, with the exception of the *M. morganii* isolate (Mm1), which appears to be less sensitive, showing an MIC of 128 mg/mL. These results are comparable to, or even better than, some previous studies that evaluated the activity of various natural products, such as plant extracts and essential oils, against bacterial species involved in diabetic wound infections, as reported in many recent reviews [64, 65, 66, 67]. Furthermore, Tsaffo et al. [4] noted MICs ranging from 64 to 1024 *μ*g/mL for aqueous and various hydroethanolic extracts of *Eriosema robustum* leaf against various bacteria isolated from diabetic wounds in Dschang, Cameroon, including *K. pneumoniae*, *P. aeruginosa*, and *S. aureus*. Furthermore, the findings of this research align with those of Simo et al. [22], which demonstrated the antibacterial activity of oil from *F. margarita* liver extracted by exudation, with MICs ranging from 16 to 256 mg/mL against bacterial strains responsible for food poisoning, including *E. coli*, *K. pneumoniae*, *P. aeruginosa*, and *S. aureus*. They noted that this oil is either bactericidal or bacteriostatic, depending on the species and strain. This antibacterial activity could be attributed to the presence of certain fatty acids in these oils, known for their antimicrobial properties, as noted by Simo et al. [22]. Additionally, bioactive compounds such as phenolics and carotenoids, present in substantial amounts in the *F. margarita* liver, as noted in the present study, could also explain this antibacterial activity, given their known antimicrobial properties through various mechanisms. Furthermore, other compounds found in *F. margarita* liver, as in other fish oils, such as sterols, liposoluble vitamins, and minerals [17], could also contribute to this antibacterial activity. Indeed, sterols are known to exhibit antimicrobial activity primarily by disrupting microbial cell membranes, inhibiting key enzymes involved in microbial metabolism, interfering with nutrient uptake, and inducing oxidative stress [68, 69]. Liposoluble vitamins, such as vitamin E, exhibit antimicrobial activity through several mechanisms, including the inhibition of bacterial protein lipocalins (which are involved in resistance to antibiotics) and the induction of oxidative stress [70]. Likewise, dietary minerals exhibit antimicrobial activity through various mechanisms, including disrupting microbial cell membranes, inhibiting key enzymes necessary for microbial metabolism, generating ROS to induce oxidative stress, and competing with microbes for essential nutrients [71].

Incorporating spice powders prior to the extraction of *F. margarita* liver oil generally enhanced antibacterial activity across all bacterial species, as demonstrated in Table 4. Specifically, compared to oil extracted without spices, the MICs decreased in 35 cases, remained unchanged in 24 cases, and increased in only one case. Consequently, the interaction between oil and spices was synergistic in 58.33% of cases, additive in 40.00% of cases, and indifferent in 1.67% of cases, with no antagonistic interactions observed. Spices contain bioactive compounds such as phenolics and carotenoids that can enhance the antibacterial properties of the oil. These compounds may work synergistically with the oil's natural components to inhibit bacterial growth more effectively. The slight increase in MICs in one case could be due to specific interactions between the spice and the bacterial strain that reduced the oil's efficacy. The combination of various phenolic compounds and carotenoids in these oils likely creates a synergistic effect, where the combined action of these bioactive compounds is greater than the sum of their individual effects, enhancing the overall antibacterial activity.

Oils extracted with *M. myristica* and *Z. officinale* showed the most significant improvements, exhibiting synergistic interactions in 80% of the isolates tested and additive interactions in 20% of the cases. *A. sativum*-extracted oil demonstrated moderate improvement, with synergistic interactions in 40% of the isolates and additive interactions in 60%. In contrast, *P. nigrum*-extracted oil showed the least improvement, with indifferent interactions in 1.67% of the isolates, additive interactions in 60%, and synergistic interactions in 33.33%. These results can be explained by the bioactive compound content of the different oils. These compounds were higher in oils extracted with *M. myristica* and *Z. officinale* compared to *A. sativum*-extracted and *P. nigrum*-extracted oils. The higher TPC and flavonoid content in spiced oils suggest that these oils have a greater concentration of these bioactive compounds, enhancing their ability to inhibit bacterial growth. Likewise, the higher carotenoid content in spiced oils suggests that these compounds are better preserved during the extraction process when spices are used, enhancing the oil's antimicrobial effectiveness.

These findings suggest that incorporating spices during the extraction process can enhance the bioactive compound content of oils, potentially developing them into natural antibacterial agents. This approach could be particularly beneficial for combating bacteria with high antibiotic resistance, such as those involved in diabetic wound infections.

#### 3.3.3. Antioxidant Activity of Extracted Oil

**Figure 2:**
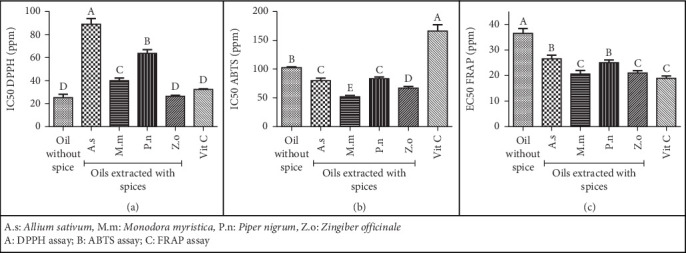
Antioxidant activity of *Fontitrygon margarita* liver oil. (a) DPPH assay; (b) ABTS assay; (c) FRAP assay.

Figure 2 illustrates the antioxidant capacity of *F. margarita* liver oil through various assays: DPPH (Figure 2a), ABTS (Figure 2b), and FRAP (Figure 2c). For the DPPH assay, the oil extracted without spices exhibited good scavenging activity (IC50 30.45 ppm), though it was lower than vitamin C (IC50 12.03 ppm). Adding spices improved the scavenging activity, reducing the IC50 by 12.67%–27.15%, with *M. myristica* extract showing the lowest IC50 (18.88 ppm), followed by *Z. officinale* and *P. nigrum* (20.17–20.72 ppm) and *A. sativum* with the highest IC50 (22.63 ppm). Likewise, the oil with no spices had good scavenging activity in the ABTS assay (IC50 16.61 ppm), comparable to vitamin C (16.06 ppm). The addition of *A. sativum* and *P. nigrum* did not significantly affect the activity (IC50 15.70–16.26 ppm), while *Z. officinale* and *M. myristica* enhanced the activity, reducing the IC50 by 10.41%–19.06%. In the FRAP assay, the oil without spices showed good reducing power (EC50 30.45 ppm), though lower than vitamin C (EC50 18.88 ppm). Adding spices generally improved the reducing power, decreasing the EC50 by 12.87%–28.77%, with *Z. officinale* and *M. myristica* showing the best improvement (EC50 21.69–21.90 ppm) compared to *A. sativum* and *P. nigrum* (23.54–26.53 ppm). These results align with previous findings on the carotenoid content of fish oils, where spices helped prevent carotenoid oxidation, demonstrating their antioxidant activity.

The antioxidant properties of *F. margarita* liver oil without spices can be attributed to its natural bioactive compounds, such as phenolics, flavonoids, and carotenoids, which neutralize free radicals and reduce oxidative stress. Other compounds like sterols, liposoluble vitamins, and minerals [17] may also contribute. Incorporating spices during extraction enhances the oil's antioxidant activity by increasing bioactive compound content, creating synergistic effects, and leveraging the specific contributions of different spices. This results in a more potent natural antioxidant agent, beneficial for managing oxidative stress-related conditions.

Overall, incorporating spices during the extraction process can enhance the antioxidant properties of *F. margarita* liver oil, making it a more effective natural antioxidant agent. These enhanced properties make the spiced oils a promising natural therapeutic option for managing diabetic wound infections, potentially improving healing outcomes and reducing complications associated with oxidative stress.

## 4. Conclusion

This study underscores the burden of diabetic wound infections and the challenge of antibiotic resistance. It identifies *S. aureus*, *K. pneumoniae*, and *E. coli* as common pathogens. Imipenem and gentamicin were effective against many isolates, but careful management is needed to prevent resistance. The high rate of polymicrobial infections complicates treatment, highlighting the need for comprehensive microbial profiling and alternative treatments like *F. margarita* liver oil. This oil, especially when extracted with spices like *M. myristica* and *Z. officinale*, shows promise as a natural antibacterial and antioxidant agent. These enhanced properties make the spiced oils a promising natural therapeutic option for managing diabetic wound infections, potentially improving healing outcomes and reducing complications associated with bacterial infections and oxidative stress. Future research should focus on understanding the antibacterial and antioxidant mechanisms of *F. margarita* liver oil. Evaluating its wound healing efficacy through clinical trials is crucial. Developing stable, potent formulations and conducting long-term studies will enhance its clinical utility and impact on diabetic wound healing.

## Figures and Tables

**Table 1:** Influence of sociodemographic and clinical factors on diabetic wound infection.

	**n**	**Statute, ** **n** **(%)**		**Chi** ^**2**^	*p*
		**Noninfected**	**Infected**		
Sociodemographic characteristics					
Sex					
Male	47	10 (21.28%)	37 (78.72%)	1.92	0.166
Female	31	11 (35.48%)	20 (64.57%)		
Age					
< 41	9	5 (55.56%)	4 (44.44%)	6.48	0.039
41–60	29	4 (13.79%)	25 (86.21%)		
> 60	40	12 (30.00%)	28 (70.00%)		
Clinical factors					
Type of diabetes					
Type 1	10	5 (50.00%)	5 (50.00%)	3.11	0.078
Type 2	68	16 (23.53%)	52 (76.47%)		
Comorbidities					
Yes	47	11 (23.40%)	36 (76.60%)	0.74	0.388
None	31	10 (32.26%)	21 (67.74%)		
Wound location					
Thigh	8	6 (75.00%)	2 (25.00%)	32.47	< 0.0001
Back	1	1 (100%)	0 (0%)		
Buttock	10	7 (70.00%)	3 (30.00%)		
Hand	11	4 (36.36%)	7 (63.64%)		
Foot	48	3 (6.25%)	45 (93.75%)		
Mechanism of wound					
During washing	5	4 (80.00%)	1 (20.00%)	26.62	0.0001
During medical care	10	8 (80.00%)	2 (20.00%)		
Spontaneous	33	5 (15.15%)	28 (84.85%)		
Trauma	30	4 (13.33%)	26 (86.67%)		
Wound macroscopy					
Necrosis	24	4 (16.67%)	20 (83.33%)	61.06	< 0.0001
Suppuration	10	0 (0%)	10 (100%)		
Necrosis and suppuration	27	0 (0%)	27 (100%)		
No signs of necrosis and suppuration	17	17 (100%)	0 (0%)		

**Table 2:** Different bacteria isolated from the 58 infected wounds.

**Isolated bacteria**		**Frequency**				
		**Wound topography**				**Total, ** **n** ** (%)**
**Species**	**Characteristics**	**Foot (** **n** **)**	**Hand (** **n** **)**	**Buttock (** **n** **)**	**Thigh (** **n** **)**	
Different isolates						
*Staphylococcus aureus*	Gram-positive cocci	23	7	0	1	**31 (54.38%)**
*Klebsiella pneumoniae*	Gram-negative bacilli	22	1	1	0	**23 (40.35%)**
*Escherichia coli*	Gram-negative bacilli	14	2	0	1	**17 (29.82%)**
*Proteus mirabilis*	Gram-negative bacilli	6	0	0	0	**6 (10.53%)**
*Streptococcus beta-hemolyticus*	Gram-positive cocci	3	0	2	0	**5 (8.77%)**
*Pseudomonas aeruginosa*	Gram-negative bacilli	4	0	1	0	**5 (8.77%)**
*Morganella morganii*	Gram-negative bacilli	2	2	0	0	**4 (7.02%)**
*Enterobacter aerogenes*	Gram-negative bacilli	2	0	0	1	**3 (5.26%)**
*Staphylococcus epidermidis*	Gram-positive cocci	3	0	0	0	**3 (5.26%)**
*Acinetobacter* spp.	Gram-negative cocci	1	0	0	0	**1 (1.75%)**
Association of different bacterium isolates						
No association						
*Staphylococcus aureus*		0	3	0	0	**3 (5.26%)**
*Klebsiella pneumoniae*		9	0	0	0	**9 (15.79%)**
*Escherichia coli*		1	0	0	1	**2 (3.51%)**
*Enterobacter aerogenes*		1	0	0	0	**1 (1.75%)**
*Streptococcus beta-hemolyticus*		0	0	1	0	**1 (1.75%)**
*Pseudomonas aeruginosa*		2	0	1	0	**3 (5.26%)**
Total		**13**	**3**	**2**	**1**	**19 (33.33%)**
Bimicrobial association						
*Staphylococcus aureus + Escherichia coli*		8	1	0	0	**9 (15.79%)**
*Staphylococcus aureus + Enterobacter aerogenes*		1	0	0	1	**2 (3.51%)**
*Staphylococcus aureus + Morganella morganii*		1	2	0	0	**3 (5.26%)**
*Staphylococcus aureus + Klebsiella pneumoniae*		4	0	0	0	**4 (7.02%)**
*Staphylococcus aureus + Proteus mirabilis*		4	0	0	0	**4 (7.02%)**
*Staphylococcus aureus + Pseudomonas aeruginosa*		2	0	0	0	**2 (3.51%)**
*Staphylococcus aureus + Acinetobacter* spp.		1	0	0	0	**1 (1.75%)**
*Staphylococcus epidermidis + Klebsiella pneumoniae*		2	0	0	0	**2 (3.51%)**
*Staphylococcus epidermidis + Morganella morganii*		1	0	0	0	**1 (1.75%)**
*Streptococcus beta-hemolyticus + Klebsiella pneumoniae*		1	0	1	0	**2 (3.51%)**
*Escherichia coli + Streptococcus beta-hemolyticus*		1	0	0	0	**1 (1.75%)**
*Escherichia coli + Klebsiella pneumoniae*		2	0	0	0	**2 (3.51%)**
*Klebsiella pneumoniae + Proteus mirabilis*		2	0	0	0	**2 (3.51%)**
Total		**30**	**3**	**1**	**1**	**35 (61.41%)**
Trimicrobial association						
*Staphylococcus aureus + Escherichia coli + Streptococcus beta-hemolyticus*		1	0	0	0	1 (1.75%)
*Staphylococcus aureus + Escherichia coli + Klebsiella pneumoniae*		1	1	0	0	2 (3.51%)
Total		**2**	**1**	**0**	**0**	**3 (5.26%)**

*Note:* Data in bold is related to the total value.

**Table 3:** Susceptibility of bacterial isolates to the tested antibiotics.

**Bacteria**		**Antibiotic**	**Susceptibility, ** **n** ** (%)**		
**Species**	**N**		**Susceptible**	**Intermediate**	**Resistant**
Sensitivity of different bacterial isolates					
*Staphylococcus aureus*	31	Amoxicillin–clavulanic acid	23 (74.19%)	5 (16.12%)	3 (9.68%)
		Ciprofloxacin	20 (64.52%)	6 (19.35%)	5 (16.12%)
		Gentamicin	28 (90.32%)	0	3 (9.68%)
		Imipenem	31 (100%)	0	0
*Klebsiella pneumoniae*	23	Amoxicillin–clavulanic acid	5 (21.74%)	3 (9.68%)	15 (65.22%)
		Ciprofloxacin	13 (56.52%)	9 (39.13%)	1 (4.35%)
		Gentamicin	17 (73.91%)	3 (13.04%)	3 (13.04%)
		Imipenem	20 (86.96%)	0	3 (13.04%)
*Escherichia coli*	17	Amoxicillin–clavulanic acid	6 (35.29%)	0	11 (64.71%)
		Ciprofloxacin	10 (58.82%)	6 (35.29%)	1 (5.88%)
		Gentamicin	14 (82.35%)	0	3 (17.65%)
		Imipenem	12 (70.59%)	2 (11.76%)	3 (17.65%)
*Proteus mirabilis*	6	Amoxicillin–clavulanic acid	1 (16.66%)	3 (50%)	2 (33.33%)
		Ciprofloxacin	3 (50%)	2 (33.33%)	1 (16.66%)
		Gentamicin	5 (83.33%)	0	1 (16.66%)
		Imipenem	4 (66.66%)	1 (16.66%)	1 (16.66%)
*Streptococcus beta-hemolyticus*	5	Amoxicillin–clavulanic acid	4 (80%)	0	1 (20%)
		Ciprofloxacin	4 (80%)	1 (20%)	0
		Gentamicin	0	1 (20%)	4 (80%)
		Imipenem	5 (100%)	0	0
*Pseudomonas aeruginosa*	5	Amoxicillin–clavulanic acid	0	0	5 (100%)
		Ciprofloxacin	1 (20%)	3 (60%)	1 (20%)
		Gentamicin	1 (20%)	1 (20%)	3 (60%)
		Imipenem	2 (40%)	0	3 (60%)
*Morganella morganii*	4	Amoxicillin–clavulanic acid	0	0	4 (100%)
		Ciprofloxacin	2 (50%)	1 (25%)	1 (25%)
		Gentamicin	3 (75%)	0	1 (25%)
		Imipenem	1 (25%)	0	3 (75%)
*Enterobacter aerogenes*	3	Amoxicillin–clavulanic acid	0	0	3 (100%)
		Ciprofloxacin	2 (66.66%)	0	1 (33.33%)
		Gentamicin	2 (66.66%)	0	1 (33.33%)
		Imipenem	3 (100%)	0	0
*Staphylococcus epidermidis*	3	Amoxicillin–clavulanic acid	2 (66.66%)	0	1 (33.33%)
		Ciprofloxacin	1 (33.33%)	0	2 (66.66%)
		Gentamicin	3 (100%)	0	0
		Imipenem	3 (100%)	0	0
*Acinetobacter* spp.	1	Amoxicillin–clavulanic acid	0	0	1 (100%)
		Ciprofloxacin	1 (100%)	0	0
		Gentamicin	1 (100%)	0	0
		Imipenem	1 (100%)	0	0

Global sensitivity to antibiotics					
Total bacterial isolates					
	98	Amoxicillin–clavulanic acid	41 (41.84%)	11 (11.22%)	46 (46.94%)
		Ciprofloxacin	58 (59.18%)	27 (27.55%)	13 (13.27%)
		Gentamicin	74 (75.51%)	5 (5.10%)	19 (19.39%)
		Imipenem	81 (82.65%)	4 (4.08%)	13 (13.27%)
According to Gram type					
Gram-positive	39	Amoxicillin–clavulanic acid	29 (74.36%)	5 (12.82%)	5 (12.82%)
		Ciprofloxacin	25 (64.10%)	7 (17.95%)	7 (17.95%)
		Gentamicin	31 (79.49%)	1 (2.56%)	7 (17.95%)
		Imipenem	39 (100%)	0	0
Gram-negative	59	Amoxicillin–clavulanic acid	12 (20.33%)	6 (10.17%)	41 (69.49%)
		Ciprofloxacin	33 (55.93%)	20 (33.89%)	6 (10.17%)
		Gentamicin	43 (72.88%)	4 (6.77%)	12 (20.33%)
		Imipenem	42 (71.18%)	4 (6.77%)	13 (22.03%)

**Table 4:** Antibacterial activity of *Fontitrygon margarita* liver oil against multiresistant bacterial isolates.

**Bacterial isolates**			**MIC (mg/mL)**			
**Species**	**Code**	**Extracted without spices**	**Extracted with spices**			
			***A.s***	***M.m***	***P.n***	***Z.o***
*Escherichia coli*	Ec1	32	16^Sy^	16^Sy^	16^Sy^	16^Sy^
	Ec2	64	64^Ad^	32^Sy^	32^Sy^	16^Sy^
	Ec3	64	32^Sy^	64^Ad^	64^Ad^	32^Sy^

*Klebsiella pneumoniae*	Kp1	64	32^Sy^	32^Sy^	64^Ad^	32^Sy^
	Kp2	32	32^Ad^	32^Ad^	32^Ad^	32^Ad^
	Kp3	64	64^Ad^	32^Sy^	64^Ad^	64^Ad^

*Pseudomonas aeruginosa*	Pa1	64	64^Ad^	64^Ad^	64^Ad^	32^Sy^
	Pa2	32	16^Sy^	16^Sy^	32^Sy^	16^Sy^
	Pa3	64	32^Sy^	32^Sy^	64^Ad^	32^Sy^

*Staphylococcus aureus*	Sa1	32	16^Sy^	16^Sy^	32^Sy^	8^Sy^
	Sa2	64	64^Ad^	32^Sy^	32^Sy^	32^Sy^
	Sa3	16	16^Ad^	8^Sy^	32^In^	16^Ad^

*Enterobacter aerogenes*	Ea1	64	64^Ad^	32^Sy^	64^Ad^	32^Sy^

*Morganella morganii*	Mm1	128	128^Ad^	64^Sy^	128^Ad^	32^Sy^

*Proteus mirabilis*	Pm1	32	32^Ad^	8^Sy^	32^Ad^	16^Sy^

*Note:* Interaction (Ad, additive; An, antagonism; In, indifference; Sy, synergy).

Abbreviations: *A.s*, *Allium sativum*; *M.m*, *Monodora myristica*; *P.n*, *Piper nigrum*; *Z.o*, *Zingiber officinale*.

## Data Availability

The data used to support the findings of this study are available from the corresponding author upon request.
